# Association between Hepatitis B virus infection and liver metastasis in colorectal cancer

**DOI:** 10.1002/mco2.584

**Published:** 2024-06-17

**Authors:** Chenqin Le, Chengcheng Liu, Bin Lu, Xinbin Zhou, Yeernaer Jiamaliding, Tian Jin, Siqi Dai, Jun Li, Kefeng Ding, Qian Xiao

**Affiliations:** ^1^ Department of Colorectal Surgery and Oncology (Key Laboratory of Cancer Prevention and Intervention China National Ministry of Education) The Second Affiliated Hospital Zhejiang University School of Medicine Hangzhou China; ^2^ Center for Medical Research and Innovation in Digestive System Tumors Ministry of Education Hangzhou China; ^3^ Zhejiang Provincial Clinical Research Center for CANCER Hangzhou China; ^4^ Cancer Center of Zhejiang University Hangzhou China; ^5^ Nursing Department The Second Affiliated Hospital of Zhejiang University School of Medicine Hangzhou China

**Keywords:** colorectal cancer, hepatitis B virus infection, liver metastasis

## Abstract

The association between Hepatitis B virus (HBV) infection and colorectal liver metastases (CRLM) remains ambiguous in current population‐based evidence. To clarify this, we present a retrospective analysis of 5871 colorectal cancer (CRC) patients. Propensity score matching (PSM) was applied to harmonize age and sex disparities within HBV+ (*n* = 1696) and HBV‐ (*n* = 4175) groups and further within HBV+ subgroups of chronic (CHB, *n* = 474) and occult (OHB, *n* = 1222) infections. Our initial results indicated a significant association between HBV infection and synchronous colorectal liver metastasis (SYN‐CRLM); however, this association dissipated after PSM was employed to adjust for confounding variables. No significant association was observed between HBV infection and metachronous colorectal liver metastases (MET‐CRLM) both before and after PSM. Further analysis revealed that HBV replication status did not influence the incidence of CRLM. However, HBV+ participants demonstrated an increased incidence of metachronous extrahepatic metastases, particularly to the lungs. Our findings imply that neither past nor present HBV infection is significantly correlated with the occurrence of SYN‐CRLM or MET‐CRLM. The absence of an association between HBV replication status and CRLM incidence highlights the importance of incorporating a broader range of factors in the clinical management of CRLM beyond the status of HBV infection.

## Graphical abstract

In a retrospective cohort of 5871 colorectal cancer patients, we aimed to investigate the association between both previous and current Hepatitis B virus (HBV) infections and colorectal liver metastasis (CRLM). Propensity score matching (PSM) was applied to harmonize age and sex disparities within HBV+ (n = 1696) and HBV‐ (n = 4175) groups and further within HBV+ subgroups of chronic (CHB, n = 474) and occult (OHB, n = 1222) infections. We observed that null association between HBV infection status and synchronous CRLM in participants after PSM. Furthermore, there was no significant association between HBV infection and metachronous CRLM.

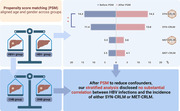

## 1 INTRODUCTION

Hepatitis B virus (HBV) infection is a well‐established driver of primary liver cancer development. The 2020 Interim Report of the China Primary Liver Cancer Clinical Registration Survey revealed that 83.77% of primary liver cancers in China originated from HBV infection, underscoring its critical role in liver cancer pathogenesis. However, the link between HBV infection and secondary liver cancer, specifically colorectal cancer (colorectal liver metastasis [CRLM]), remains unclear. Given that 15%–25% of patients with colorectal cancer (CRC) present with secondary liver cancer at diagnosis and considering the lethality of liver metastasis in CRC, understanding the potential correlation between HBV and CRLM could have significant clinical implications, such as identifying high‐risk patients and informing treatment strategies.

The relationship between HBV infection and CRLM is intricate and mainly involves cellular and molecular mechanisms. The “seed and soil” hypothesis suggests that a liver affected by HBV might be less conducive to the metastatic spread of CRC.^1^ Chronic HBV infection often causes liver fibrosis and vascular alterations, hindering the access of metastatic cells to the liver. Moreover, enhanced expression of adhesion molecules, such as PECAM‐1 and integrin receptors, in hepatic sinusoidal endothelial cells may impede metastatic cell adherence and infiltration.^2^ Concurrently, HBV's immunomodulatory effects involving activated cytotoxic T lymphocytes and Kupffer cells can help suppress CRLM by targeting metastatic cells. Conversely, a few studies have suggested mechanisms by which HBV can promote CRLM, such as increased levels of MCP‐1, which may enhance CRC growth, and elevated chemokines linked to CRC metastasis.^3^ Several studies have highlighted an increased expression of chemokines such as CCL20, CXCL6, and CXCL9/10/11 in patients infected with HBV.^4^, ^5^ Intriguingly, these chemokines are not only associated with CRC metastasis but might also play a pivotal role in the onset of CRC.

However, population‐based evidence shows that the association between HBV infection and CRLM is controversial. Liu et al.’s meta‐analysis, which included ten studies, indicated that HBV could potentially serve as a protective factor against CRLM. A previous study found that the incidence of CRLM in HBV‐infected patients was remarkably reduced (odds ratio [OR]: 0.51, 95% confidence interval [CI]: 0.28–0.91).^6^ However, these results should be interpreted with caution because of the apparent heterogeneity. Recently, evidence from cohort studies has been accumulating. Qiu et al. found that chronic hepatitis B (CHB), inactive HBsAg carriers (IC), and resolved hepatitis B (RHB), were related to a lower incidence of liver metastasis.^7^ Furthermore, Zhao et al. showed that HBV infection was associated with fewer and smaller liver metastases and a lower recurrence rate among patients diagnosed with CRLM undergoing hepatectomy.^8^ However, based on a retrospective cross‐sectional study of over 4,000 patients with CRC, Huo et al. found that HBV infection may promote the occurrence of synchronous (SYN) liver metastasis in patients with CRC.^9^ Similarly, in the past 2 years, several retrospective analyses have suggested that HBV replication may contribute to the development of CRLM, whereas liver fibrosis may act as a protective factor against CRLM.^10^, ^11^ These key observations underscore the inherent contradictions in the current body of literature, highlighting the need for large‐sample studies with rigorous controls for confounding factors.

To address this gap, we conducted a retrospective study including 5871 patients diagnosed with CRC at the Second Affiliated Hospital of Zhejiang University School of Medicine between January 2013 and December 2021. Using propensity score matching (PSM), we matched the baseline data of the HBV infection and non‐infection groups and investigated the association between HBV infection and synchronous colorectal liver metastasis (SYN‐CRLM) and metachronous colorectal liver metastases (MET‐CRLM). Furthermore, the association between HBV infection status and extrahepatic metastases was explored.

## 2 RESULTS

### 2.1 Baseline characteristics

Among the included 5871 patients with CRC, 1,696 (28.89%) were identified as the HBV+ group, including 474 patients with CHB and 1,222 patients with occult HBV infection (OHB). Analysis of the baseline characteristics, detailed in Table 1, showed a statistically significant difference in sex distribution, with the HBV+ group presenting a higher proportion of males (*p *= 0.001). However, no significant differences were observed between the HBV+ and HBV‐ groups in terms of age, primary CRC location, tumor size, histological grade, and TNM classification.

**TABLE 1:** Baseline characteristics of colorectal cancer (CRC) in Hepatitis B virus‐infected (HBV+) and Hepatitis B virus‐uninfected (HBV–) groups.

	Before PSM			After PSM		
Characteristic	HBV+ (*n* = 1696)	HBV− (*n* = 4175)	*p*‐Value	HBV+ (*n* = 1695)	HBV− (*n* = 1695)	*p*‐Value
Sex			0.001			1
Male	1084 (63.9)	2475 (59.3)		1084 (64.0)	1084 (64.0)	
Female	612 (36.1)	1700 (40.7)		611 (36.0)	611 (36.0)	
Age, y, mean ± SD	62.00 ± 12.31	62.53 ± 12.63	0.151	61.98 ± 12.29	62.00 ± 12.29	0.965
Primary CRC						
Location			0.640			0.894
Right‐sided colon	294 (27.63)	772 (28.76)		293 (27.56)	295 (27.44)	
Left‐sided colon	303 (28.48)	778 (28.99)		251 (23.61)	263 (24.47)	
Rectum	467 (43.89)	1134 (42.25)		519 (48.82)	517 (48.09)	
Tumor size, cm, mean ± SD	4.25 ± 2.56	4.14 ± 2.36	0.501	4.25 ± 2.56	4.06 ± 1.94	0.301
Grade			0.303			0.157
Poor	151 (12.7)	302 (11.5)		151 (12.7)	117 (10.7)	
Moderate/Well	1040 (87.3)	2334 (88.5)		1040 (87.3)	978 (89.3)	
T stage			0.433			1
TIS‐T2	280 (22.0)	582 (20.8)		280 (22.0)	252 (22.0)	
T3‐T4	994 (78.0)	2211 (79.2)		994 (78.0)	895 (78.0)	
N stage			0.830			0.58
N0	699 (55.3)	1529 (54.9)		699 (55.3)	621 (54.1)	
N1‐N2	565 (44.7)	1257 (45.1)		565 (44.7)	527 (45.9)	
SYN‐CRLM	242 (14.3)	475(11.4)	0.002	242 (14.3)	220 (13.0)	0.298

*Notes*: *p‐*Value: significance level—a *p*‐value less than 0.05 typically indicates statistical significance.

Abbreviations: CRC, colorectal cancer; HBV‐, hepatitis B virus uninfected; HBV+, hepatitis B virus infection; PSM, propensity score matching; SD, standard deviation; SYN‐CRLM, synchronous colorectal liver metastasis; y, years.

Further subgroup analysis revealed that the CHB subgroup was diagnosed with CRC at a younger age than the OHB subgroup (*p *< 0.001). In contrast, the two HBV+ subgroups were well balanced in primary CRC location, tumor size, grade, and TNM staging (*p *> 0.05) (Table S1).

### 2.2 Balancing groups by PSM

To reduce confounding biases, we balanced the differences in baseline characteristics between the two groups using PSM, matched the HBV+ and HBV‐ groups 1:1 according to sex and age, and included 3390 patients. Notably, our initial baseline data revealed no significant differences between the two groups in TNM stage, tumor size, differentiation grade, or primary CRC location (Table 1). Consequently, these variables were excluded from the matching criteria. Post‐matching, the baseline characteristics were well‐balanced between the two groups (Table 1).

In the HBV+ subgroup analysis, PSM was applied to the status of the CHB and OHB groups among CRC patients with HBV infection. The matching process resulted in 474 matched pairs, totaling 948 patients, again based on sex and age. This refined matching within the HBV+ subgroup and ensured a rigorous comparison between the CHB and OHB groups, facilitating a more accurate assessment of HBV infection status on CRC outcomes (Table S1).

### 2.3 Association between HBV and CRLM

Our analysis revealed an increased prevalence of SYN‐CRLM in the HBV+ group compared with the HBV‐ group. Before PSM, the prevalence was 14.3% for the HBV+ group (242/1,696) and 11.4% for the HBV‐ group (475/4,175) (*p *= 0.002; Table 1). Univariate logistic regression analysis showed that CRC patients with HBV+ were associated with higher SYN‐CRLM (OR = 1.296, 95% CI: 1.098–1.531; *p *= 0.002; Table 2). When further adjusted for CA199, CEA, AFP, ALT, AST, GGT, ALP, ALB, AG, TBIL, DBIL, IBIL, LDH, PLT, and FIB‐4, the significant association persisted with an adjusted OR (aOR) of 0.732 (95% CI: 0.602–0.890; *p *= 0.002; Table 2). After PSM, the prevalence of SYN‐CRLM in the HBV+ group was maintained at 14.3% (242/1,695), whereas in the HBV‐ group, it was 13.0% (220/1695). Multivariate logistic regression analysis showed a null association between HBV infection and SYN‐CRLM with an aOR of 1.208 (95% CI: 0.954–1.530; *p *= 0.116; Table 2). More details are shown in Table S2.

**TABLE 2:** Effect of hepatitis B virus (HBV) infection on synchronous colorectal liver metastasis (SYN‐CRLM) and metachronous (MET)‐CRLM before and after propensity score matching (PSM).

SYN‐CRLM				
	Before PSM(*N* = 5871)		After PSM (*N* = 3190)	
	**Crude model**	**Multivariable Model**	**Crude model**	**Multivariable Model**
HBV‐	Ref	Ref	Ref	Ref
HBV+_a_	1.296 (1.098–1.531)	0.732 (0.602–0.890)	0.896 (0.736–1.090)	1.208 (0.954–1.530)
*p‐*Value	0.002	0.002	0.271	0.116

**MET‐CRLM**				
	**Before PSM (*N* = 5871)**		**After PSM (*N* = 3190)**	
	**Crude model**	**Multivariable Model**	**Crude model**	**Multivariable Model**
HBV‐	Ref	Ref	Ref	Ref
HBV+_b_	1.050 (0.749∼1.471)	0.957 (0.672∼1.363)	1.155 (0.780∼1.710)	0.863 (0.574∼1.298)
*p‐*Value	0.778	0.808	0.471	0.479

*Note*: *p‐*Value: Significance level—A *p‐*value less than 0.05 typically indicates statistical significance.

HBV+ indicates individuals with evidence of hepatitis B virus infection, which includes patients with chronic hepatitis B (CHB) and those with occult hepatitis B infection (OHB).

HBV‐ refers to individuals without any detectable markers of hepatitis B virus infection, implying no current or past evidence of HBV infection in the bloodstream.

Abbreviations: MET‐CRLM, metachronous colorectal liver metastasis; PSM, propensity score matching; SYN‐CRLM, synchronous colorectal liver metastasis.

^a^ OR (95% CI) denotes the odds ratio, which is the ratio of the odds of an event occurring in the HBV+ group to the odds of it occurring in the HBV‐ group. The 95% confidence interval indicates that we can be 95% certain that the true value of the odds ratio lies between the lower and upper bounds provided.

^b^ RR (95% CI) signifies the relative risk, which compares the risk of an event occurring in the HBV+ group to the risk in the HBV‐ group. The 95% confidence interval for the relative risk provides a range within which the true relative risk is expected to fall with 95% confidence.

We further investigated the association between HBV infection and MET‐CRLM after excluding CRC patients diagnosed with SYN‐CRLM. In the pre‐PSM dataset, the accumulative proportions of MET‐CRLM were 3.30% and 3.46% in the HBV+ and HBV‐ groups, respectively, with a relative risk (RR) of 1.05 (95% CI: 0.749–1.471). When we further adjusted for CA199, CEA, AFP, ALT, AST, GGT, ALP, ALB, AG, TBIL, DBIL, IBIL, LDH, PLT, and FIB‐4, the null association remained between HBV infection status and MET‐CRLM with an RR of 0.957 (95% CI: 0.672−1.363; *p *= 0.808; Table 2). After PSM, the MET‐CRLM cumulative prevalence for the HBV+ group was 3.55%, while the HBV‐ group showed a higher prevalence of 4.07%. Multivariate logistic regression analysis showed a non‐significant association between HBV infection status and MET‐CRLM with an RR of 0.863 (95% CI: 0.574–1.298; *p *= 0.479; Table 2). Table S3 shows the determinants of the MET‐CRLM after incorporating HBV and additional variables.

### 2.4 Association between CHB and CRLM

As for CRC patients with positive HBV, when compared with CRC patients with OHB infection, a minor and non‐significant higher prevalence of SYN‐CRLM was found among CRC patients with CHB infection with an OR of 1.066 (95% CI: 0.78–1.448; *p *= 0.684). Similar associations were identified in the multivariable analysis with an aOR of 0.749 (95% CI: 0.507–1.105; *p *= 0.145). After PSM, the findings remained statistically insignificant, with a univariate OR of 0.832 (95% CI: 0.581–1.191; *p *= 0.316) and a multivariate aOR of 0.686 (95% CI: 0.436–1.079; *p *= 0.103), as detailed in Table 3. More details can be found in Table S4.

**TABLE 3:** Subgroup analysis of synchronous colorectal liver metastasis (SYN‐CRLM) and metachronous colorectal liver metastasis (MET‐CRLM) based on current and past hepatitis B virus (HBV) infection statuses before and after propensity score matching (PSM).

SYN‐CRLM				
	Before PSM (*N* = 1696)		After PSM (*N* = 948)	
	**Crude model**	**Multivariable Model**	**Crude model**	**Multivariable Model**
OHB	Ref	Ref	Ref	Ref
CHB_a_	1.066 (0.785–1.448)	0.749 (0.507–1.105)	0.832 (0.581–1.191)	0.686 (0.436–1.079)
*p‐*Value	0.684	0.145	0.316	0.103

**MET‐CRLM**				
	**Before PSM (*N* = 1696)**		**After PSM (*N* = 948)**	
	**Crude model**	**Multivariable Model**	**Crude model**	**Multivariable Model**
OHB	Ref	Ref	Ref	Ref
CHB_b_	1.489 (0.736–3.013)	0.763 (0.368–1.581)	2.044 (0.946–4.415)	0.616 (0.273–1.391)
*p‐*Value	0.268	0.467	0.069	0.244

*Notes*: *p‐*Value: Significance level—A *p‐*value less than 0.05 typically indicates statistical significance.

CHB: chronic hepatitis B; a long‐term infection with the hepatitis B Virus characterized by the persistent presence of hepatitis B surface antigen (HBsAg) for more than six months.

OHB: occult hepatitis B infection; a form of HBV infection where hepatitis B core antibody (HBcAb) or hepatitis B e antibody (HBeAb) are present, but the hepatitis B surface antigen (HBsAg) is not detectable.

Abbreviations: MET‐CRLM, metachronous colorectal liver metastasis; PSM, propensity score matching; SYN‐CRLM, synchronous colorectal liver metastasis.

^a^ OR (95% CI) denotes the odds ratio, which is the ratio of the odds of an event occurring in the HBV+ group to the odds of it occurring in the HBV‐ group. The 95% confidence interval indicates that we can be 95% certain that the true value of the odds ratio lies between the lower and upper bounds provided.

^b^ RR (95% CI) signifies the relative risk, which compares the risk of an event occurring in the HBV+ group to the risk in the HBV‐ group. The 95% confidence interval for the relative risk provides a range within which the true relative risk is expected to fall with 95% confidence.

As for MET‐CRLM, before PSM, compared with CRC patients with OHB, results showed a non‐statistically increased accumulative prevalence in CRC patients with CHB with an RR of 1.489 (95% CI: 0.736–3.013; *p *= 0.268; Table 3). However, when we further adjusted for CA199, CEA, AFP, ALT, AST, GGT, ALP, ALB, AG, TBIL, DBIL, IBIL, LDH, PLT, and FIB‐4, MET‐CRLM, CRC patients with CHB were non‐statistically lower in numbers than CRC patients with OHB infection with an RR of 0.763 (95% CI: 0.368–1.581; Table 3). After PSM, the RR between infection type of HBV presented similar patterns in the univariate analysis (RR = 2.044, 95% CI: 0.946–4.415; *p *= 0.069; Table 3) and multivariate analysis (aRR = 0.616, 95% CI: 0.273–1.391; *p *= 0.244; Table 3). More details are shown in Table S5.

### 2.5 Influence of HBV replication status on the prevalence of SYN‐CRLM and MET‐CRLM

In addition, we investigated the association between HBV replication status by categorizing the patients into HBsAg+/HBeAg+ (*n* = 22) and HBsAg+/HBeAg‐ (*n* = 431) subgroups. The prevalence of SYN‐CRLM was 27.3% in the HBsAg+/HBeAg+ patients, while the prevalence was 19.3% in HBsAg+/HBeAg‐ patients (*p *= 0.517). Regarding MET‐CRLM, only ten cases were identified in HBsAg+/HBeAg‐ patients. This detailed examination, as presented in Table 4, underscores that HBeAg status, a marker of active HBV replication, does not significantly influence the risk of SYN‐CRLM or MET‐CRLM in CHB patients.

**TABLE 4:** Prevalence of colorectal liver metastasis (CRLM) in hepatitis B surface antigen (HBsAg)+ patients with or without HBeAg+.

	HBsAg+/HBeAg+ (*N* = 22)	HBsAg+/HBeAg– (*N* = 431)	*p‐*Value
SYN‐CRLM	6 (27.3)	83 (19.3)	0.517
MET‐CRLM	0 (0)	10 (2.3)	1.000

*Notes*: *p‐*Value: Significance level—A *p‐*value less than 0.05 typically indicates statistical significance.

Abbreviations: MET‐CRLM, metachronous colorectal liver metastasis; SYN‐CRLM, synchronous colorectal liver metastasis.

### 2.6 Association between HBV and extrahepatic metastases

When we limited the analysis to the CRC patients after PSM between the HBV+ (n = 1695) and HBV‐ (n = 1695) groups, the accumulative prevalence in the HBV+ group was 7.32% for SYN‐extrahepatic metastases (Mets), which was non‐significantly higher than that in the HBV‐ group (5.72%) (*p *= 0.0603; Table 5). Similar patterns were observed for specific metastatic locations such as lung, peritoneal, omental, ovarian, bone, retroperitoneal lymph nodes, and intracranial regions, without significance according to the status of HBV infection.

**TABLE 5:** Distribution of extrahepatic metastases in hepatitis B Virus (HBV)+ and HBV– groups.

Group	HBV+ (*N* = 1695)	HBV− (*N* = 1695)	*p‐*Value
**SYN‐Extrahepatic Mets**	124 (7.32)	97 (5.72)	0.0603
SYN Lung Mets	80 (4.72)	61 (3.60)	0.1022
SYN Peritoneal Mets	10 (0.59)	6 (0.35)	0.3162
SYN Omental Mets	4 (0.24)	1 (0.06)	0.1794
SYN Ovarian Mets	9 (0.53)	7 (0.41)	0.6162
SYN Bone Mets	13 (0.77)	10 (0.59)	0.5302
SYN Retroperitoneal Lymph Node Mets	4 (0.24)	7 (0.41)	0.3649
SYN Intracranial Mets	4 (0.24)	5 (0.29)	0.7385
**MET‐Extrahepatic Mets**	134 (7.91)	79 (4.66)	**< 0.0001**
MET Lung Mets	91 (5.37)	56 (3.30)	**0.0032**
MET Peritoneal Mets	6 (0.35)	5 (0.29)	0.7627
MET Omental Mets	1 (0.06)	1 (0.06)	1.0000
MET Ovarian Mets	6 (0.35)	4 (0.24)	0.5265
MET Bone Mets	17 (1.00)	9 (0.53)	0.1153
MET Retroperitoneal Lymph Node Mets	6 (0.35)	1 (0.06)	0.0585
MET Intracranial Mets	7 (0.41)	3 (0.18)	0.2052

*Notes*: *p‐*Value: Significance level—A *P* value less than 0.05 typically indicates statistical significance.

Abbreviations: HBV‐, hepatitis B virus uninfected; HBV+, hepatitis B virus‐infected; MET, metachronous; Mets, metastases; SYN, synchronous.

In contrast, there were significantly increased metachronous extrahepatic metastases in the HBV+ group (7.91%) compared with those in the HBV‐ group (4.66%, *p *< 0.0001; Table 5). Particularly, significant differences in the accumulative prevalence were pronounced for metachronous lung metastases, wherein HBV+ patients exhibited a prevalence of 5.37% versus 3.30% in HBV‐ patients (*p *= 0.0032; Table 5).

## 3 DISCUSSION

Liver metastasis is a common and serious complication of CRC. The role of HBV infection in CRLM has been debated, with some studies suggesting that it may be protective, whereas others indicate that it could promote CRLM. To address these conflicting views, we conducted a large retrospective cohort study and utilized PSM to balance the confounding factors. Our study found that neither current nor past HBV infections were significantly associated with synchronous or MET‐CRLM.

Previous studies have provided conflicting evidence regarding the role of HBV infection in the development of CRLM. While several studies advocate HBV as a protective factor, emphasizing its role in enhancing liver immunity and promoting apoptosis in tumor cells,^12^, ^13^, ^14^ others posit an opposing view. These studies suggest that HBV can facilitate CRLM through pathways similar to those observed in primary liver cancer,^3^, ^15^ with recent large‐scale analyses supporting this hypothesis.^9^, ^10^, ^11^, ^16^ To our knowledge, the present study is the largest retrospective cohort analysis investigating the effects of HBV infection on liver metastasis in CRC.

Our study takes a different approach from that of documented reports as we hypothesized that patients with existing or past HBV infection may undergo a process leading to liver damage, whereby the virus is inserted into viral DNA and triggers an immune response that creates an immunotolerant microenvironment. Therefore, we included patients with CHB and OHB in the HBV+ group and patients with total HBV marker (HBVM) negative in the HBV‐ group.

China has a high prevalence of HBV infection, estimated at 5%–6% for HBsAg in the general population.^17^ The prevalence of HBsAg in patients with CRC included in our study was 8.07% (474/5871), higher than the national average. This raises the following question: does HBV infection affect primary lesions in CRC? In support of this hypothesis, an analysis of data from 500,000 individuals in the Kadoorie Biobank in China 2019 by Song et al. found a positive correlation between HBV infection and CRC, which was further supported by Liu et al.’s prospective study in 2021.^18^ Although not detailed in the results section of the present study, Table S6 provides additional data on age differences observed between the HBsAg+ and HBsAg‐ groups, highlighting a trend toward earlier onset of CRC in HBsAg+ patients, consistent with findings from previous studies.^9^

Surprisingly, contrary to the prevailing perspectives in the literature, our research suggests no significant association between HBV infection and the risk of SYN‐CRLM or MET‐CRLM, highlighting the complexity of this relationship. Our study embarked on a sequential, layered investigation into the role of HBV infection in CRLM, starting with a broad analysis of HBV+ versus HBV‐ patients and progressively narrowing to more specific subgroups within the HBV+ cohort. This structured approach allowed us to dissect the complexities of HBV's influence on CRLM, providing a holistic understanding of its potential impact. Initially, our analysis suggested a potential association between HBV infection and CRLM; however, the significance of this relationship was attenuated after accounting for confounding factors through PSM. This nuanced finding indicates that other variables, such as age and sex, may play a more pronounced role in influencing the risk of liver metastasis. Previous studies have suggested that liver metastases are more likely to be detected in younger patients with CRC, possibly because of differences in follow‐up or genetic factors.^19^ Additionally, the protective effect of endogenous estrogen may explain the lower incidence of CRLM in female patients.^20^

For a more detailed analysis of the HBV+ group, we employed PSM to rigorously compare the CHB and OHB subgroups and found no significant difference in the risk of CRLM. This careful subdivision within the HBV+ population allowed for a nuanced analysis, which revealed that HBV replication status, as indicated by HBeAg positivity, does not significantly influence the likelihood of developing SYN‐CRLM or MET‐CRLM. Diverging from prior research that predominantly focused on synchronous CRLM,^9^ our comprehensive approach evaluated both synchronous and metachronous CRLM outcomes, and this broadened analysis allowed us to conclude that HBV replication status does not exert a significant influence on the development of CRLM, a finding consistent across all stages of liver metastasis. Our findings, which demonstrate a lack of significant association across different stages of liver metastasis, contribute a novel perspective to the body of research and underscore the importance of considering a range of factors beyond HBV replication in the clinical management of CRC.

Interestingly, our study revealed a significantly higher incidence of metachronous extrahepatic metastases, particularly lung metastases, in the HBV+ group when compared with the HBV‐ group, which aligns with the conclusions drawn by Huo et al..^9^ This observation, which emerged after meticulous PSM to equilibrate confounding variables, challenges the conventional understanding of HBV's role in CRC progression. While the prevalence of synchronous extrahepatic metastases did not differ significantly between the two groups, the marked increase in metachronous metastases, particularly in the lungs, merits attention.

Our findings have important implications for patient counseling and management, particularly in areas with a high prevalence of HBV infection. Clinicians often grapple with the potential risk of CRLM in patients with current or previous HBV infection. The evidence from our study provides a measure of reassurance, indicating that HBV infection, whether active or resolved, is not associated with an increased risk of either synchronous or metachronous CRLM. Consequently, our results suggest that standard surveillance protocols should be sufficient for patients with CRC, irrespective of their HBV status, without the need for additional measures specifically aimed at detecting liver metastasis. Furthermore, the heightened rate of metachronous lung metastases observed in the HBV+ group underlines the importance of vigilant postoperative surveillance. This finding advocates for a nuanced approach to follow‐up care, one that is cognizant of the potential for late‐onset metastases in CRC patients. However, our findings do not support the necessity for differential follow‐up strategies based solely on HBV infection status. Instead, we call for a uniform application of clinical guidelines that ensure that all patients receive consistent monitoring, with attention to individual clinical scenarios that may warrant closer observation.

Our study, while offering valuable insights into the association between HBV infection and CRC prognosis, has some limitations. First, the retrospective design inherently introduces bias and may lack the depth or control achievable in prospective cohort studies, particularly because we relied on historical data exclusively sourced from the Second Affiliated Hospital of the Zhejiang University School of Medicine. This could lead to unaccounted confounders that may have skewed our findings and thus curtail their broader applicability across diverse geographic or demographic settings. Our study did not consider certain risk factors, such as the impact of sex and hormone levels on immune responses, which could potentially affect the interaction between HBV and CRLM.

Despite the challenges inherent to a single‐center retrospective analysis, our study employed rigorous statistical methodologies and provided a comprehensive review of patient data. These results challenge the established understanding of the relationship between HBV and CRLM, underscoring the need for further investigations into the cellular and molecular mechanisms involved. Future research should explore the molecular pathways influenced by HBV that may affect CRLM and offer potential therapeutic avenues. To enhance the robustness and applicability of our insights, conducting multicenter studies across diverse populations and initiating prospective studies are imperative to elucidate the effects and mechanisms of HBV infection in CRLM.

## 4 CONCLUSION

In conclusion, our study indicates that neither previous nor current HBV infections are significantly linked to the occurrence of SYN‐CRLM or MET‐CRLM, nor does the replication status of HBV notably influence CRLM risk. Intriguingly, we observed that HBV infections might escalate the risk of metachronous extrahepatic metastases, notably in promoting lung metastases. These findings underscore the complexity of HBV's role in CRC progression and highlight the necessity for prospective studies to further explore the implications of HBV infection on CRLM development.

## 5 METHODS

### 5.1 Patient selection

This study was conducted following the ethical standards of the 1964 Declaration of Helsinki and approved by The Ethics Committee of the Second Affiliated Hospital of Zhejiang University School of Medicine (approval number: 2023‐0009). Given the retrospective nature of the study, the committee waived the requirement for informed consent.

**Inclusion Criteria**: Patients diagnosed with CRC at the Second Affiliated Hospital of Zhejiang University School of Medicine between January 2013 and December 2021.

**Exclusion Criteria**: Patients lacking HBsAg information.

From hospital records, 7159 patients were initially identified as diagnosed with CRC. After applying the exclusion criteria, 1288 patients were excluded owing to the absence of HBsAg information. Finally, 5871 patients were included in the study.

### 5.2 Clinical data

Clinical data were collected in three categories: (1) Epidemiological data, including sex and age; (2) pathological data, including tumor location, tumor size, pathological grade, and TNM stage; and (3) serological data: HBV serological markers (HBsAg, HBsAb, HBeAg, HBeAb, HBcAb, HBV‐DNA, CA199, CEA, AFP, ALT, AST, GGT, ALP, ALB, AG, TBIL, DBIL, IBIL, LDH, PLT, and FIB‐4). All serological data used in this analysis were within 30 days before or after the day the patient was pathologically diagnosed with CRC.

### 5.3 Diagnostic criteria for HBV infection and CRLM

According to the patient's HBVM and HBV DNA, the cases were preliminarily divided into two groups: HBV infection (HBV+) and HBV‐uninfected (HBV‐). The HBV+ group included patients with CHB (including HBsAg‐positive patients) and OHB (including HBcAb‐ or HBeAb‐positive patients who tested negative for HBsAg).

The definitions of simultaneous and metachronous CRLM differ in the literature. In this study, we defined patients with an interval shorter than or equal to 6 months from the first diagnosis of CRC as having SYN‐CRLM and those with an interval longer than 6 months as having metachronous CRLM (MET‐CRLM). The study flowchart can be found in Supporting Information as Figure S1.

### 5.4 Propensity score matching

Propensity scores were calculated using a logistic regression model, wherein the dependent variable was the group membership (HBV+ or HBV‐), and the independent variables were age and sex. These scores represented the probability of being in the HBV+ group based on the observed covariates. Patients from the HBV+ group were matched 1:1 with patients from the HBV‐ group based on nearest neighbor matching without replacement, using a caliper width of 0.2 standard deviations of the logit of the propensity score. After matching, we assessed the balance between the two groups by comparing the standardized differences in all covariates. A standardized difference below 0.1 for a given covariate indicated a negligible difference in the mean or prevalence of that covariate between the two matched groups.

Following this initial matching, an additional layer of PSM was conducted within the HBV+ group to compare CHB and OHB patients. This analysis employed identical matching parameters—nearest neighbor matching without replacement and a caliper width of 0.2 standard deviations of the logit of the propensity score—focusing again on age and sex as the critical covariates for matching.

### 5.5 Logistic regression analysis

Before and after PSM, both univariate and multivariate logistic regression analyses were performed to evaluate the association between HBV infection and SYN‐CRLM and MET‐CRLM outcomes. In the multivariate logistic regression analysis, all variables, including HBV infection, CA199, CEA, AFP, ALT, AST, GGT, ALP, ALB, AG, TBIL, DBIL, IBIL, LDH, PLT, and FIB‐4, were entered simultaneously to identify significant predictors of SYN‐CRLM and MET‐CRLM. The same logistic regression approach was applied to the matched CHB and OHB subgroups within the HBV+ cohort.

### 5.6 Post‐hoc power analysis

In our study, to validate the strength of our findings, we executed a post hoc power analysis, focusing on the observed differences in liver metastasis rates between the HBV+ (20.2%) and HBV‐ (17.1%) groups. We established an α error probability (Type I error) at 0.05, signifying a 5% likelihood of erroneously rejecting the true null hypothesis. Based on this formula, the post hoc power was calculated at approximately 0.93. This suggests a 93% probability that our study would aptly discern an effect, given the observed disparity in liver metastasis rates. Our analyses were two‐tailed, allowing for potential effects in both directions. The effect size was gauged by the noticeable rate difference between the groups, which was 3.1%.

Power=Φ(Zα/2−n×p(1−p)∣p1−p2∣)

### 5.7 Statistical analysis

SPSS 22 (IBM) was used for statistical analysis. Continuous variables were compared using the Student's t‐test, while categorical data were evaluated using the chi‐square test. Statistical significance was set at a two‐tailed *p*‐value < 0.05.

## AUTHOR CONTRIBUTIONS

All authors contributed to the study's conception and design. *Conceptualization*: Chenqin Le, Kefeng Ding and Qian Xiao. *Methodology*: Chenqin Le and Qian Xiao. *Investigation*: Chenqin Le, Chengcheng Liu, Bin Lu, Xinbin Zhou, Yeernaer Jiamaliding, Tian Jin, Siqi Dai, Jun Li, Kefeng Ding and Qian Xiao. *Writing—Original Draft*: Chenqin Le. *Writing—Review & Editing*: Chenqin Le, Kefeng Ding and Qian Xiao. *Funding Acquisition*: Kefeng Ding, Qian Xiao and Chenqin Le. *Supervision*: Kefeng Ding and Qian Xiao. All authors have read and approved the final manuscript.

## CONFLICT OF INTEREST STATEMENT

The authors declare no conflict of interest.

## ETHICS STATEMENT

The Ethics Committee of the Second Affiliated Hospital of Zhejiang University School of Medicine (approval number: 2023‐0009). Because this was a retrospective study, the committee waived the requirement for informed consent.

## Supporting information

Table Table Table Table 

Supporting Information

## Data Availability

Data supporting the findings of this study are available from the corresponding author upon request.
