# Supplementary figures and images for: Association between Hepatitis B virus infection and liver metastasis in colorectal cancer
Source: MedComm (2020). 2024 Jun 17;5(7):e584. doi: 10.1002/mco2.584 (PMC11181900; doi:10.1002/mco2.584)

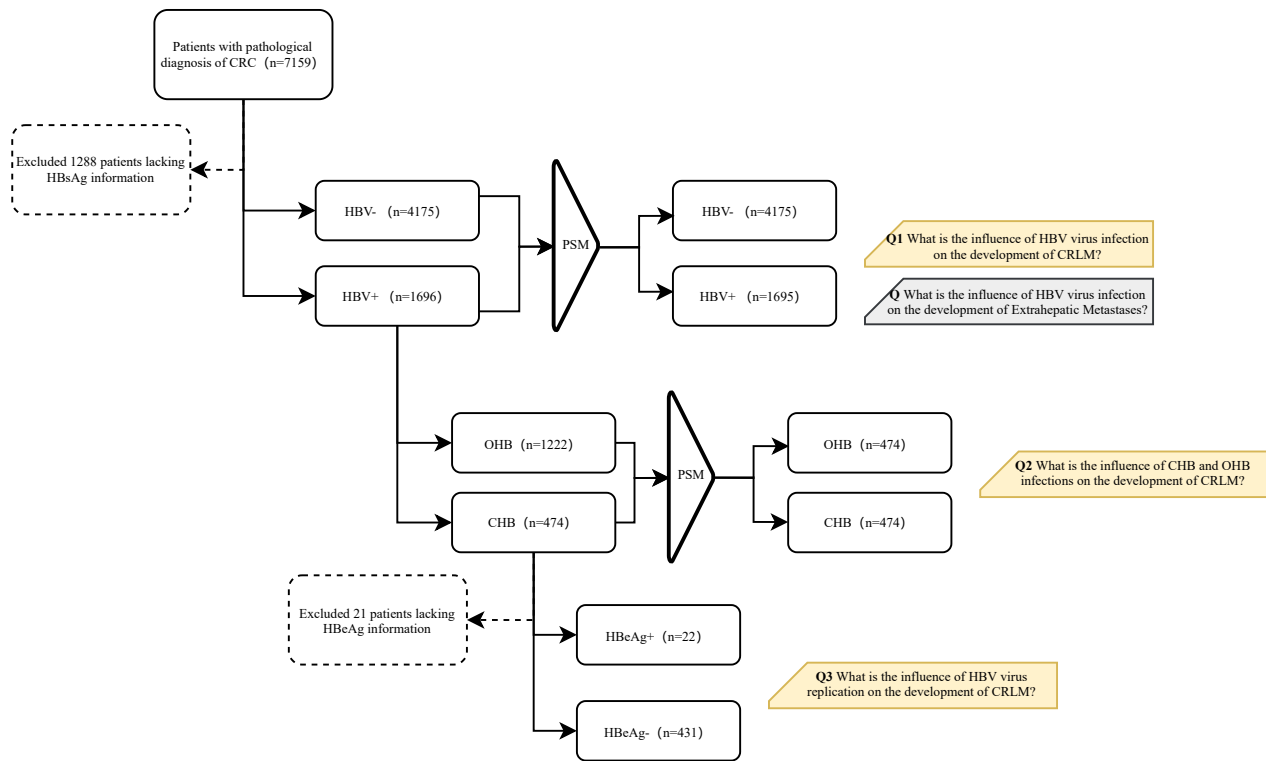

Supplement: Supplementary file 1 — Table Table Table Table [file MCO2-5-e584-s001.pdf]
