# Supplementary material for: Association between Hepatitis B virus infection and liver metastasis in colorectal cancer
Source: MedComm (2020). 2024 Jun 17;5(7):e584. doi: 10.1002/mco2.584 (PMC11181900; doi:10.1002/mco2.584)
Supplement: Supplementary file 2 — Supporting Information [file MCO2-5-e584-s002.docx]

**Association between Hepatitis B Virus Infection and Liver Metastasis in Colorectal Cancer**

**Running title: HBV infection and liver metastasis in CRC**

Chenqin Le^a,b,c,d, 1^, Chengcheng Liu ^a,b,c,d,1^, Bin Lu^e^, Xinbin Zhou^e^, Yeernaer Jiamaliding ^a,b,c,d^, Tian Jin ^a,b,c,d^, Siqi Dai ^a,b,c,d^, Jun Li ^a,b,c,d^, Kefeng Ding ^a,b,c,d^ *, Qian Xiao ^a,b,c,d^ *

^a^Department of Colorectal Surgery and Oncology (Key Laboratory of Cancer Prevention and Intervention, China National Ministry of Education), The Second Affiliated Hospital, Zhejiang University School of Medicine, Hangzhou, Zhejiang, China

^b^Center for Medical Research and Innovation in Digestive System Tumors, Ministry of Education, China

^c^Zhejiang Provincial Clinical Research Center for CANCER, China

^d^Cancer Center of Zhejiang University, Hangzhou, Zhejiang, 310058, China

^e^Nursing Department, The Second Affiliated Hospital of Zhejiang University School of Medicine, Hangzhou, Zhejiang, China

(^1^ denotes first authors)

**Corresponding author: Qian Xiao*

*E-mail: qxiao3@zju.edu.cn*

*Kefeng Ding*

*E-mail:* [*dingkefeng@zju.edu.cn*](mailto:dingkefeng@zju.edu.cn)

**Table S1. Baseline Characteristics of CRC in CHB and OHB Groups**

| Characteristic | Before PSM | | | After PSM | | |
| --- | --- | --- | --- | --- | --- | --- |
|  | CHB(N=474) | OHB(N=1222) | *P* Value | CHB(N=474) | OHBN=474) | *P* Value |
| **Sex** |  |  | 0.103 |  |  | 0.894 |
| Male | 288 (60.8) | 796 (65.1) |  | 288 (60.8) | 285 (60.1) |  |
| Female | 186 (39.2) | 426 (34.9) |  | 186 (39.2) | 189 (39.9) |  |
| **Age, y, mean±SD** | 58.67 (11.7) | 63.32 (12.23) | <0.001 | 58.67 (11.71) | 58.70 (11.80) | 0.969 |
| **Primary CRC** |  |  |  |  |  |  |
| **Location** |  |  | 0.958 |  |  | 0.492 |
| Right-sided colon | 87 (27.80) | 207 (27.56) |  | 87 (27.80) | 91 (32.27) |  |
| Left-sided colon | 72 (23.00) | 179 (23.83) |  | 72 (23.00) | 61 (21.63) |  |
| Rectum | 154 (49.20) | 365(48.60) |  | 154 (49.20) | 130 (46.10) |  |
| **Tumor size, cm, mean±SD** | 4.21 (1.93) | 4.26 (2.78) | 0.878 | 4.21 (1.93) | 4.58 (3.81) | 0.421 |
| **Grade** |  |  |  |  |  |  |
| Poor | 45 (14.4) | 106 (12.1) | 0.341 | 45 (14.4) | 46 (13.6) | 0.854 |
| Moderate/Well | 268 (85.6) | 772 (87.9) |  | 268 (85.6) | 293 (86.4) |  |
| **T stage** |  |  |  |  |  |  |
| TIS-T2 | 72 (22.0) | 208 (22.0) | 1.000 | 72 (22.0) | 81 (22.3) | 0.997 |
| T3-T4 | 256 (78.0) | 738 (78.0) |  | 256 (78.0) | 283 (77.7) |  |
| **N stage** |  |  |  |  |  |  |
| N0 | 179 (55.1) | 520 (55.4) | 0.977 | 179 (55.1) | 196 (54.6) | 0.961 |
| N1-N2 | 146 (44.9) | 419 (44.6) |  | 146 (44.9) | 163 (45.4) |  |
| **SYN-CRLM** | 65 (13.7) | 177 (14.5) | 0.741 | 65 (13.7) | 76 (16.0) | 0.361 |

**Notes:**
*P* value: significance level - a *P* value less than 0.05 typically indicates statistical significance.

**Abbreviations:**
CRC: colorectal cancer; CHB: chronic hepatitis B; HBV+: hepatitis B virus infection; HBV-: hepatitis B virus uninfected; OHB: occult hepatitis B; PSM: propensity score matching; SYN-CRLM, synchronous colorectal liver metastasis; y, years; SD: standard deviation.

**Table S2. Multivariate Logistic Regression Analysis for SYN-CRLM Before and After Propensity-Score Matching**

| Factors | Before PSM (N=5,871） | | | After PSM (N=3,190) | | |
| --- | --- | --- | --- | --- | --- | --- |
|  | aOR | (95% CI） | *P* value | aOR | (95% CI） | *P* value |
| HB | 0.732 | 0.602~0.890 | 0.002 | 1.208 | 0.954~1.530 | 0.116 |
| CA199 | 1.000 | 1.000~1.001 | 0.000 | 1.000 | 1.000~1.001 | 0.000 |
| CEA | 1.002 | 1.001~1.003 | 0.000 | 1.002 | 1.001~1.003 | 0.000 |
| AFP | 1.002 | 1.000~1.004 | 0.086 | 1.001 | 0.999~1.003 | 0.461 |
| ALT | 1.011 | 1.001~1.021 | 0.026 | 1.006 | 0.992~1.019 | 0.396 |
| AST | 0.975 | 0.960~0.991 | 0.002 | 0.983 | 0.964~1.002 | 0.077 |
| GGT | 1.003 | 1.001~1.005 | 0.006 | 1.005 | 1.002~1.008 | 0.000 |
| ALP | 1.003 | 1.000~1.006 | 0.024 | 1.003 | 1.000~1.007 | 0.077 |
| ALB | 1.052 | 1.029~1.076 | 0.000 | 1.033 | 1.005~1.063 | 0.022 |
| AG | 0.590 | 0.379~0.919 | 0.019 | 0.797 | 0.461~1.375 | 0.414 |
| TBIL | 1.005 | 0.998~1.013 | 0.156 | 1.007 | 0.998~1.016 | 0.108 |
| DBIL | 0.899 | 0.850~0.952 | 0.000 | 0.847 | 0.782~0.919 | 0.000 |
| IBIL | 0.990 | 0.965~1.017 | 0.471 | 0.995 | 0.962~1.029 | 0.756 |
| LDH | 1.004 | 1.003~1.005 | 0.000 | 1.005 | 1.003~1.006 | 0.000 |
| PLT | 1.002 | 1.000~1.003 | 0.015 | 1.002 | 1.000~1.004 | 0.042 |
| FIB4 | 1.157 | 1.054~1.271 | 0.002 | 1.102 | 0.978~1.242 | 0.110 |

**Notes:**
*P* value: significance level - a *P* value less than 0.05 typically indicates statistical significance.
aOR: adjusted odds of an event occurring relative to the odds of not occurring, considering potential confounders.

(95% CI): 95% confidence interval - an interval estimate that we are 95% confident that the true odds ratio falls within the range.

**Abbreviations:**

AFP, alpha-fetoprotein; ALB, albumin; ALP, alkaline phosphatase; ALT, alanine aminotransferase; AST, aspartate aminotransferase; CA199, carbohydrate antigen 199; CEA, carcinoembryonic antigen; DBIL, direct bilirubin; FIB4, fibrosis index based on the 4 factors; GGT, γ-glutamyltransferase; HBV, hepatitis B virus; LDH, lactate dehydrogenase; PLT, platelet; SYN-CRLM: synchronous colorectal liver metastasis; TBIL, total bilirubin.

**Table S3. Multivariate Logistic Regression Analysis for MET-CRLM Before and After Propensity-Score Matching**

| Factors | Before PSM (N=5,871） | | | After PSM (N=3,190) | | |
| --- | --- | --- | --- | --- | --- | --- |
|  | aOR | (95% CI） | *P* value | aOR | (95% CI） | *P* value |
| HB | 0.957 | 0.672~1.363 | 0.808 | 0.863 | 0.574~1.298 | 0.479 |
| CA199 | 1.000 | 1.000~1.000 | 0.918 | 1.000 | 0.999~1.000 | 0.899 |
| CEA | 1.000 | 0.999~1.001 | 0.458 | 1.001 | 1.000~1.002 | 0.270 |
| AFP | 1.003 | 1.000~1.006 | 0.038 | 1.003 | 1.000~1.006 | 0.032 |
| ALT | 1.007 | 0.988~1.026 | 0.495 | 1.006 | 0.981~1.032 | 0.629 |
| AST | 0.984 | 0.955~1.014 | 0.292 | 0.994 | 0.958~1.031 | 0.736 |
| GGT | 1.001 | 0.997~1.005 | 0.755 | 0.996 | 0.990~1.003 | 0.289 |
| ALP | 1.000 | 0.995~1.006 | 0.937 | 1.002 | 0.996~1.008 | 0.518 |
| ALB | 0.986 | 0.967~1.006 | 0.166 | 0.980 | 0.951~1.010 | 0.199 |
| AG | 3.060 | 1.694~5.525 | 0.000 | 2.209 | 0.985~4.953 | 0.054 |
| TBIL | 0.992 | 0.971~1.013 | 0.439 | 0.990 | 0.963~1.017 | 0.448 |
| DBIL | 0.995 | 0.949~1.045 | 0.854 | 1.038 | 0.955~1.127 | 0.384 |
| IBIL | 1.014 | 0.972~1.057 | 0.523 | 1.033 | 0.984~1.084 | 0.191 |
| LDH | 0.997 | 0.993~1.002 | 0.221 | 0.998 | 0.992~1.003 | 0.371 |
| PLT | 1.002 | 1.000~1.004 | 0.081 | 1.003 | 0.999~1.006 | 0.129 |
| FIB4 | 1.123 | 0.967~1.304 | 0.129 | 1.016 | 0.782~1.320 | 0.906 |

**Notes:**
*P* value: significance level - a *P* value less than 0.05 typically indicates statistical significance.
aOR (adjusted odds ratio): adjusted odds of an event occurring relative to the odds of not occurring, considering potential confounders.

(95% CI): 95% confidence interval - an interval estimate that we are 95% confident that the true odds ratio falls within the range.

**Abbreviations:**

AFP, alpha-fetoprotein; ALB, albumin; ALP, alkaline phosphatase; ALT, alanine aminotransferase; AST, aspartate aminotransferase; CA199, carbohydrate antigen 199; CEA, carcinoembryonic antigen; DBIL, direct bilirubin; FIB4, fibrosis index based on the 4 factors; GGT, γ-glutamyltransferase; HBV, hepatitis B virus; LDH, lactate dehydrogenase; PLT, platelet; MET-CRLM: metachronous colorectal liver metastasis; TBIL, total bilirubin.

**Table S4. Multivariate Logistic Regression Analysis for SYN-CRLM Before and After Propensity-Score Matching**

| Factors | Before PSM (N=1,696） | | | After PSM (N=948) | | |
| --- | --- | --- | --- | --- | --- | --- |
|  | aOR | (95% CI） | *P* value | aOR | (95% CI） | *P* value |
| CHB/OHB | 0.749 | 0.507~1.105 | 0.145 | 0.686 | 0.436~1.079 | 0.103 |
| CA199 | 1.001 | 1.000~1.001 | 0.000 | 1.001 | 1.000~1.001 | 0.000 |
| CEA | 1.001 | 1.000~1.002 | 0.021 | 1.002 | 1.000~1.003 | 0.037 |
| AFP | 1.002 | 0.999~1.005 | 0.244 | 1.002 | 0.999~1.005 | 0.288 |
| ALT | 1.009 | 0.990~1.028 | 0.365 | 1.004 | 0.978~1.031 | 0.759 |
| AST | 0.987 | 0.963~1.013 | 0.328 | 0.981 | 0.949~1.014 | 0.253 |
| GGT | 1.003 | 1.000~1.007 | 0.046 | 1.007 | 1.001~1.012 | 0.024 |
| ALP | 1.008 | 1.003~1.013 | 0.003 | 1.006 | 0.999~1.013 | 0.105 |
| ALB | 1.034 | 0.989~1.082 | 0.137 | 1.042 | 0.981~1.106 | 0.180 |
| AG | 0.772 | 0.350~1.704 | 0.522 | 0.606 | 0.208~1.767 | 0.359 |
| TBIL | 1.016 | 1.000~1.033 | 0.055 | 1.004 | 0.980~1.028 | 0.757 |
| DBIL | 0.838 | 0.762~0.920 | 0.000 | 0.800 | 0.692~0.926 | 0.003 |
| IBIL | 0.989 | 0.943~1.038 | 0.658 | 1.005 | 0.937~1.077 | 0.894 |
| LDH | 1.003 | 1.001~1.005 | 0.006 | 1.002 | 1.000~1.005 | 0.089 |
| PLT | 1.003 | 1.001~1.006 | 0.012 | 1.004 | 1.000~1.007 | 0.028 |
| FIB4 | 1.172 | 1.007~1.364 | 0.040 | 1.303 | 1.041~1.632 | 0.021 |

**Notes:**
*P* value: significance level - a *P* value less than 0.05 typically indicates statistical significance.
aOR (adjusted odds ratio): adjusted odds of an event occurring relative to the odds of not occurring, considering potential confounders.

(95% CI): 95% confidence interval - an interval estimate that we are 95% confident that the true odds ratio falls within the range.

**Abbreviations:**

AFP, alpha-fetoprotein; ALB, albumin; ALP, alkaline phosphatase; ALT, alanine aminotransferase; AST, aspartate aminotransferase; CA199, carbohydrate antigen 199; CEA, carcinoembryonic antigen; DBIL, direct bilirubin; FIB4, fibrosis index based on the 4 factors; GGT, γ-glutamyltransferase; HBV, hepatitis B virus; LDH, lactate dehydrogenase; PLT, platelet; SYN-CRLM: synchronous colorectal liver metastasis; TBIL, total bilirubin.

**Table S5. Multivariate Logistic Regression Analysis for MET-CRLM Before and After Propensity-Score Matching**

| Factors | Before PSM (N=1,696） | | | After PSM (N=948) | | |
| --- | --- | --- | --- | --- | --- | --- |
|  | aOR | (95% CI） | *P* value | aOR | (95% CI） | *P* value |
| CHB/OHB | 0.763 | 0.368~1.581 | 0.467 | 0.616 | 0.273~1.391 | 0.244 |
| CA199 | 1.000 | 0.999~1.001 | 0.759 | 1.000 | 0.999~1.001 | 0.712 |
| CEA | 0.999 | 0.994~1.004 | 0.723 | 0.998 | 0.991~1.005 | 0.618 |
| AFP | 0.801 | 0.612~1.049 | 0.107 | 0.856 | 0.622~1.180 | 0.343 |
| ALT | 1.022 | 0.985~1.062 | 0.249 | 1.026 | 0.975~1.079 | 0.326 |
| AST | 0.977 | 0.918~1.040 | 0.461 | 0.963 | 0.888~1.045 | 0.368 |
| GGT | 1.000 | 0.990~1.010 | 0.980 | 1.001 | 0.988~1.015 | 0.843 |
| ALP | 0.998 | 0.987~1.010 | 0.803 | 0.996 | 0.981~1.011 | 0.596 |
| ALB | 1.114 | 1.010~1.228 | 0.031 | 1.087 | 0.960~1.230 | 0.190 |
| AG | 0.471 | 0.095~2.321 | 0.355 | 0.802 | 0.110~5.822 | 0.827 |
| TBIL | 0.995 | 0.952~1.039 | 0.811 | 0.963 | 0.895~1.036 | 0.311 |
| DBIL | 1.047 | 0.911~1.204 | 0.516 | 1.021 | 0.827~1.261 | 0.848 |
| IBIL | 0.997 | 0.920~1.081 | 0.945 | 1.069 | 0.956~1.195 | 0.239 |
| LDH | 0.996 | 0.987~1.004 | 0.310 | 1.000 | 0.991~1.008 | 0.967 |
| PLT | 1.002 | 0.997~1.007 | 0.397 | 1.003 | 0.996~1.009 | 0.385 |
| FIB4 | 1.014 | 0.645~1.595 | 0.951 | 1.210 | 0.724~2.024 | 0.467 |

**Notes:**
*P* value: significance level - a *P* value less than 0.05 typically indicates statistical significance.
aOR (adjusted odds ratio): adjusted odds of an event occurring relative to the odds of not occurring, considering potential confounders.

(95% CI): 95% confidence interval - an interval estimate that we are 95% confident that the true odds ratio falls within the range.

**Abbreviations:**

AFP, alpha-fetoprotein; ALB, albumin; ALP, alkaline phosphatase; ALT, alanine aminotransferase; AST, aspartate aminotransferase; CA199, carbohydrate antigen 199; CEA, carcinoembryonic antigen; DBIL, direct bilirubin; FIB4, fibrosis index based on the four factors; GGT, γ-glutamyltransferase; HBV, hepatitis B virus; LDH, lactate dehydrogenase; PLT, platelet; MET-CRLM: metachronous colorectal liver metastasis; TBIL, total bilirubin.

**Table S6. Baseline Characteristics of CRC in HBsAg + and HBsAg - Groups**

| Characteristic | HBsAg+(N=474) | HBsAg-(N=5,397) | *P* Value |
| --- | --- | --- | --- |
| Sex |  |  | 0.987 |
| Male | 288 (60.8) | 3271 (60.6) |  |
| Female | 186 (39.2) | 2126 (39.4) |  |
| Age, y, mean±SD | 58.58±11.86 | 62.71±12.54 | <0.001 |
| Primary CRC |  |  |  |
| Location |  |  | 0.839 |
| Right-sided colon | 72(23.0) | 825(24.0) |  |
| Left-sided colon | 87（27.8) | 979（28.5） |  |
| Rectum | 154（49.2） | 1631（47.5） |  |
| Tumor size, cm, mean±SD | 4.21±1.93 | 4.16±2.45 | 0.856 |
| Grade |  |  | 0.708 |
| Poor | 38 (13.8) | 385 (12.8) |  |
| Moderate/Well | 238 (86.2) | 2627 (87.2) |  |
| T stage |  |  | 0.619 |
| TIS-T2 | 23 (12.7) | 298 (14.4) |  |
| T3-T4 | 158 (87.3) | 1778 (85.6) |  |
| N stage |  |  | 0.473 |
| N0 | 54 (31.4) | 654 (34.4) |  |
| N1-N2 | 118 (68.6) | 1246 (65.6) |  |

**Notes:**
*P* value: significance level - a *P* value less than 0.05 typically indicates statistical significance.

**Abbreviations:**
CRC: colorectal cancer; HBsAg+: positive for hepatitis B surface antigen; HBsAg-: negative for hepatitis B surface antigen; y: years; SD: standard deviation.


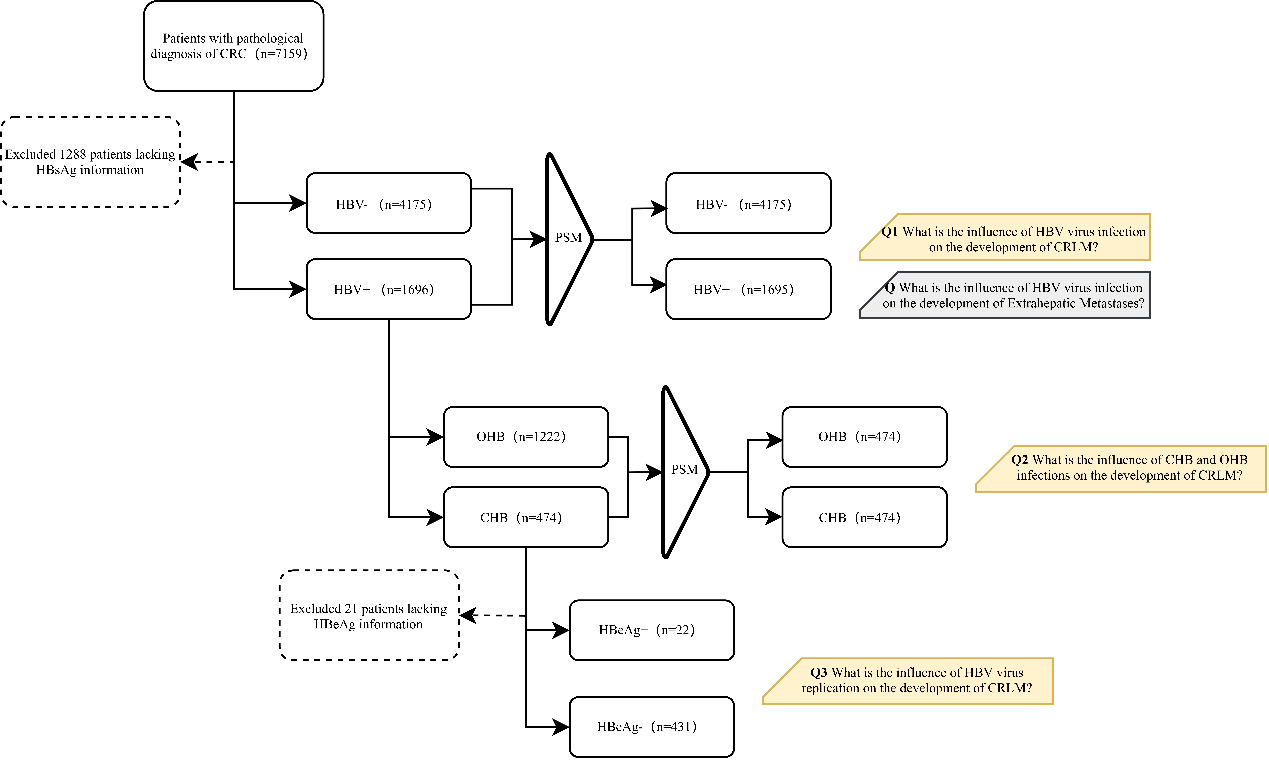


**Figure S1.** **The flow chart of the study.**

**Abbreviations:**

CHB: chronic hepatitis B virus infection group (including HBsAg-positive patients); CRC, colorectal cancer;; HBV+: hepatitis B virus infection; HBV-: hepatitis B virus uninfected; OHB: occult hepatitis B virus infection group (including HBcAb- or HBeAb-positive patients who were negative for HBsAg); PSM: propensity score matching.
